# Bedside fasciotomy under local anesthesia for acute compartment syndrome: a feasible and reliable procedure in selected cases

**DOI:** 10.1007/s10195-012-0196-9

**Published:** 2012-04-18

**Authors:** Nabil A. Ebraheim, Amr A. Abdelgawad, Molly A. Ebraheim, Sreenivasa R. Alla

**Affiliations:** 1 Department of Orthopedic Surgery, University of Toledo Medical Center, Toledo, OH USA; 2 Department of Orthopedic Surgery and Rehabilitation, Paul L. Foster School of Medicine, Texas Tech University Health Science Center, El Paso, TX USA

**Keywords:** Compartment syndrome, Bedside, Fasciotomy, Local anesthesia, Fascial release, Urgent surgery

## Abstract

**Background:**

Fasciotomy for compartment syndrome is an emergent procedure that is usually done in the operating theater under general anesthesia. Delay in performing the procedure can lead to worse outcome. Various reasons can cause delay in performing the surgery. Bedside fasciotomy under local anesthesia can be done in these cases to avoid delay in compartment release.

**Materials and methods:**

This was a retrospective study of 34 cases of acute compartment syndrome for which fasciotomy was done at the bedside under local anesthesia. The minimum follow-up period was 6 months.

**Results:**

All patients had immediate and marked improvement in pain. Thirty-three patients regained their normal muscle strength. Thirty-two patients regained normal range of motion of adjacent joints. One patient developed flexion contracture of the great toe. There was no deep infection, chronic osteomyelitis, or amputation. Superficial wound infection was noted in three patients; one patient had persistent foot drop.

**Conclusion:**

Bedside fasciotomy under local anesthesia is a feasible, safe, and effective choice for treating compartment syndrome in patients with delayed presentation or those with anticipated delay to undergo surgery in the operating theater under general or regional anesthesia. The results of this study are encouraging, as all wounds healed satisfactory and there were no cases of deep infections. The formal release of compartments in the operating room under general anesthesia continues to be the standard of care. This is the first description in the literature for bedside fasciotomy under local anesthesia with a relatively large number of patients.

## Introduction

Compartment syndrome is defined as elevation of the interstitial pressure in a closed osteofascial compartment that results in microvascular compromise [1]. As duration and magnitude of interstitial pressure increase, myoneural function is impaired, and necrosis of the soft tissues eventually develops. In 1881, Volkmann [2] recognized the association between acute ischemic events that are left untreated and late muscle contractures. Diagnosis of acute compartment syndrome depends both on clinical findings (pain out of proportion to the injury or surgery, pain with passive stretch of compartment muscles, increased narcotic requirement, tense swelling, and paresthesia) as well as on measurement of the intracompartmental pressure [3]. Previous studies show that delayed decompression of the affected compartments would lead to irreversible ischemic damage to muscles and peripheral nerves, with increased complication rate [4–8].

Fasciotomy to release the affected compartment is typically done in operating room under general or regional anesthesia after establishing the diagnosis of compartment syndrome. Fasciotomy done in the operating room is not without obstacles due to issues related to availability of the operating theater, supporting staff (nurses, surgical technicians), anesthesia, and patient readiness for surgery [3, 9]. In a recent article, Flynn et al. described compartment syndromes in children in two large pediatric trauma centers (Children’s Hospital of Philadelphia, and the Johns Hopkins Hospital in Baltimore, USA) from retrospective chart reviews [10]. The authors reported that the average time between diagnosis and fasciotomy was 2.3 h, with a range between zero (for intraoperative cases) and 8.5 h. This shows that even in the best medical centers, the time between diagnosis and fasciotomy can exceed 8 h (which means that the actual time between occurrence and fasciotomy is greater than that). Obviously, in less-well-equipped medical centers, the time between diagnosis and fasciotomy can be much longer than the time described by Flynn et al. [10].

In our department, bedside fasciotomy under local anesthesia has been performed in some cases for a certain subset of patients with compartment syndrome to avoid some of the obstacles that delay performing the fasciotomy. We conducted this retrospective review of our case series treated with this procedure to assess whether it is safe and reliable. Moreover, we here describe our indications and technique for performing this procedure.

## Materials and methods

This retrospective study was authorized by the Institutional Review Board (IRB) and conducted at our level one trauma center in compliance with the Declaration of Helsinki. We reviewed data of 347 consecutive fasciotomies from 2004 to 2007. Of these 347 patients, 34 underwent bedside fasciotomy by a single trauma surgeon. We thereafter clinically reviewed these patients and collected retrospective data: 29 patients had associated fractures (25 closed, four open) and five had soft tissue injuries. Patient characteristics are mentioned in Table 1. Table 2 shows the distribution of the involved compartments. The reasons that these 34 patients underwent bedside fasciotomy included delayed presentation of more than 6–8 h in ten cases or those with an anticipated time delay of more than 6–8 h (24 cases). The anticipated delay was due to various reasons, such as medical comorbidities needing prolonged evaluation for anesthesia (13 cases) and polytrauma patients with prolonged nonoperative resuscitation (seven cases), recent oral intake (three cases), and medical comorbidities and polytrauma (one case). Fasciotomy was done at the bedside under local anesthesia to avoid any further delay. Bedside fasciotomy was selected as an alternative to operating-room fasciotomy.

**Table 1:** Bedside fasciotomy patients

Characteristics	
Age	42.4 years
Mean in years	(16–84 years)
Sex	
Female	10
Male	24
Open fractures (11.4 %)	
Grade 1	1
Grade 2	1
Grade 3	2
Closed fractures	25
Soft tissue injuries	5
Mechanism of injury	
Motor vehicle accident	22
Fall from height	5
Simple fall	4
Pedestrian hit by vehicle	2
Assault	1

**Table 2:** Distribution and underlying injury causing acute compartment syndrome

Physical location	Underlying injury	No. of cases
Lower extremity	Tibial diaphyseal fracture	9
	Tibial plateau fracture	6
	Soft tissue injury	
	Leg	4
	Thigh	1
	Fractures of the foot	
	Lisfranc fracture	2
	Metatarsal fracture	2
	Femoral fracture	2
	Tibial pilon fracture	2
	Ankle fracture dislocation	1
Upper extremity	Forearm fracture	2
	Elbow fracture	1
	Distal radial fracture	1
	Fractures of the hand	
	Metacarpal fracture	1

Diagnosis of compartment syndrome was made on the basis of tense swelling, pain out of proportion to injury, increased narcotic requirement, pain on passive stretching of the muscles of the involved compartment, and paresthesia. Compartment pressures <30 mmHg of diastolic blood pressure were used as an adjunct to the clinical diagnosis [11]. In ten obtunded patients (from brain injury or medications), the diagnosis depended mainly on measuring the compartment pressure together with tense swelling of the compartment. Defining the exact onset of compartment syndrome is not an easy task. For patients who could describe an approximate timing of escalation of pain level, that timing was used as the onset of compartment syndrome. Patients who suffered from major injury to their extremities and then presented shortly after that to the emergency department with a clinical picture of compartment syndrome, we considered the time of injury as the time of onset of compartment syndrome.

The procedure was done at bedside in the intensive care unit, emergency department, or in the ward. Antibiotic prophylaxis was given to all patients and continued for 24 h post-operatively. The extremity was prepped and sterile sheets applied around the planned surgical field. The involved compartments were released under conscious sedation and local anesthesia. Our institutional conscious sedation protocol was midazolam (3–7 mg) and fentanyl (100–200 mcg). One percent lidocaine without epinephrine (10–30 ml) was infiltrated locally along incision line. Any subsequent procedures (e.g., fracture stabilization or debridement of open wounds) were done in the operating room after the patient was stabilized.

All cases were done by a single trauma surgeon (the senior author). Fasciotomy of the leg was done using the double-incision technique of Mubarak and Owen [12]. The anterior and lateral lower-leg compartments were released through a lateral skin incision over the lateral intermuscular septum centered halfway between the fibular shaft and the tibia crest. The two posterior compartments were accessed through a second skin incision 2 cm posterior to the medial edge of the tibia. Anterior and posterior compartments of the thigh were adequately decompressed through a single lateral incision using the technique described by Tarlow et al. [13]. The superficial and deep volar compartments of the forearm were released through a single volar incision, extending from the biceps tendon at the elbow to the center of the wrist. Dorsal decompression was performed through a straight dorsal incision [14]. Compartment syndrome of the hand and foot were managed with two longitudinal dorsal incisions over the second and fourth metacarpals and metatarsals, respectively, to release all interosseous compartments [14, 15].

Postoperative care consisted of saline-soaked wet to dry dressings on the site of fasciotomy. All fasciotomy wounds were left open initially. Wound irrigation and debridement was done as necessary. Once the swelling subsided and compartments were soft, the wounds were closed by either delayed primary closure, negative-pressure wound-therapy-assisted closure, or split-thickness skin grafting. Patients were followed after discharge at 1-week intervals until complete wound healing and thereafter every 6 weeks for a minimum of 6 months. At each follow-up visit, all patients were assessed for wound healing, infection, muscle strength, sensation, distal vascular status, adjacent joint mobility, contractures, and fracture union.

## Results

All 34 patients were followed up for a minimum of 6 months (range 6–18 months). At the time of presentation, three patients were associated with sensory deficit, one with both sensory and motor deficit (foot drop), and two with weak distal pulse. All patients had immediate and marked improvement in pain. The two patients who presented with a weak distal pulse regained their normal volume immediately after fasciotomy. Blood loss during the procedure was minimal (<100 ml). Mean procedure duration was <30 min. There were no intraoperative complications. All patients tolerated the procedure well. Wound closure was done when a healthy bed of muscle was present. Delayed primary closure was done in 14 patients and skin grafting in 20. Superficial wound infection was reported in three cases. All were treated with irrigation and debridement and with antibiotics as per culture and sensitivity. There were no deep wound infections, chronic osteomyelitis, or amputations.

Thirty-two patients had normal range of motion of adjacent joints. Two patients had decreased range of motion; however, these two patients had an associated intra-articular fracture. Of the 34 fractures, 32 achieved adequate union. There was one nonunion and one delayed union, both of which were managed appropriately. The three patients, who presented with sensory deficit over the dorsum of foot, regained their normal sensation in an average of 4 months (range 3–5 months). One patient who presented with foot drop has persistent sensory and motor deficit. One patient developed flexion contracture of the great toe.

## Discussion

The literature suggests that compartment syndrome should be treated as early as possible [4–8]. Several authors reported the results of early versus late fasciotomy: Hargens et al. [4], in a canine model, found that significant muscle necrosis occurs at an intracompartmental pressure of 30 mmHg after 8 h. Mithoefer et al. [5] showed that patients in whom the interval to decompression was more than 8 h had more long-term functional deficits. Williams et al. [6] noted that early (<12-h) fasciotomy gives better outcome and less complications compared with late (>12-h) fasciotomy. Sheridan and Masten [7] found that the complication rate for patients who had late fasciotomies was ten times greater than patients who had early fasciotomies. Ritenour et al. [8] reported results of fasciotomy in war causalities and found that patients who underwent delayed fasciotomies (in the regional medical center) had twice the major amputation rate and a threefold higher mortality rate compared with those who underwent fasciotomy in the combat theater.

We are not aware of any study that assessed the delay between diagnosis and treatment in cases of compartment syndrome. For obvious reasons, we do not think that any level one or level two trauma center will be interested in studying and assessing such delay. There were no reports in the literature regarding bedside fasciotomy for acute compartment syndrome. This is the first report of this procedure in the English literature. In our consecutive group of patients, all those treated with bedside fasciotomy were included in the study. This is not the first described emergent procedure to be done at bedside. Bedside laparotomy has been described for “patients too unstable for safe transport to the operating room” [16].

In our study group, ten patients presented after an average time delay of 8 h (range 6–10 h) following the injury, either due to delay in evacuation from the field, referral from peripheral hospitals, or patient factors (intoxicated or drug abuse). These patients had bedside fasciotomy under local anesthesia to avoid further delay in releasing the compartments. The rest of the patients (24 patients) had the procedure done at bedside because of anticipated obstacles in doing the surgery in the operating theater under general or regional anesthesia (multiple medical comorbidities necessitating prolonged preoperative evaluation, polytraumatized patients requiring prolonged nonoperative resuscitation before being transferred to the operating theater, or recent oral intake). It is extremely important to note that this procedure was done for <10 % of the patients diagnosed with compartment syndrome. The majority of cases [313 of 347 (90 %)] had the fasciotomy done in the operating theater under general or regional anesthesia.

Comparing the outcome of our method for treating compartment syndrome with the standard release in the operating room is difficult because the associated injury and the underlying diagnoses may affect the outcome of the procedure, regardless of the fasciotomy method used. Sheridan and Masten [7] described one case of infection in 22 early-operated patients (within 12 h, comparable with our group). This percentage (4.5 %) is similar to our results [three of 34 patients (9 %)]. Williams et al. [6] also had an infection rate similar to ours in their early-operated group (7.3 %). Heemskerk and Kitslaar [17] had an infection rate of 25 %; however, their patients included both trauma-induced and vascular-induced injuries, with the latter having worse outcome than the former. Comparing our results with the above studies shows that we had a similar infection rate to the standard fasciotomy done in the operating room.

The results of our study, bedsides fasciotomy for compartment syndrome, are encouraging. All wounds healed well with skin grafting or delayed primary closure. All patients regained their normal muscle strength except one, who had preoperative nerve deficit. Although three patients developed superficial wound infection, there were no cases of deep infection, chronic osteomyelitis, amputation, or death.

We would like to clarify several points related to this study: The first is that the procedure requires that the surgeon has enough experience and knowledge with the compartments that he/she is releasing in order to perform the procedure quickly, correctly, and safely. The second point is that operating room availability was not the reason for shifting from theater fasciotomy to bedside fasciotomy in any of our cases, as we are in level one trauma that has a trauma operating room available at all times. However, in other hospitals, operating room availability may be a reason for performing the fasciotomy at bedside. Another point is that >90 % of our patients undergoing fasciotomy release for compartment syndrome during the study period had their fasciotomy done in the operating theater, and by far this is still our preferred method. Also, fasciotomy under local anesthesia is not our standard of care and is only used exceptionally. Bedside fasciotomy has limitations compared with release in the operating room under general or regional anesthesia; First, concomitant fracture fixations (if needed) cannot be simultaneously performed. Second, in case muscles are found to be nonviable, debridement cannot be performed in the same session.

There were inherent limitations to our study, including the small study group, lack of a control group, and the retrospective nature of the study. The other limitation was not being able to assess the functional status of the patient after this procedure, as all patients had other limb trauma that would affect the functional status of the limb irrespective of compartment syndrome treatment.

In conclusion, bedside fasciotomy under local anesthesia is a feasible and apparently effective and safe choice for treating compartment syndrome of the limb in a small subset of patients (those with delayed presentation or in whom significant delay is anticipated before performing the procedure). The technique has some limitations and should be used only under certain circumstance. The formal release of compartments in the operating room under general anesthesia continues to be the standard of care. This is the first literature report of bedside fasciotomy in relatively large number of patients.
